# Novel Brassica hybrids with different resistance to *Leptosphaeria maculans* reveal unbalanced rDNA signal patterns

**DOI:** 10.1515/biol-2022-0032

**Published:** 2022-03-31

**Authors:** Justyna Szwarc, Janetta Niemann, Joanna Kaczmarek, Joanna Majka, Jan Bocianowski

**Affiliations:** Department of Genetics and Plant Breeding, Poznań University of Life Sciences, Dojazd 11, 60-632 Poznan, Poland; Department of Genetics and Plant Breeding, Poznań University of Life Sciences, Dojazd 11, 60-632 Poznan, Poland; Institute of Plant Genetics of the Polish Academy of Sciences, Strzeszynska 34, 60-479 Poznan, Poland; Institute of Experimental Botany, Czech Acad Sci, Centre of the Region Haná for Biotechnological and Agricultural Research, Šlechtitelů 31, 77900 Olomouc, Czech Republic; Institute of Plant Genetics of the Polish Academy of Sciences, Strzeszynska 34, 60-479 Poznan, Poland; Department of Mathematical and Statistical Methods, Poznan University of Life Sciences, Wojska Polskiego 28, 60-637 Poznan, Poland

**Keywords:** *Brassica*, FISH, blackleg, field resistance

## Abstract

Hybridization of *Brassica napus* with various Brassicaceae species can result in obtaining new forms with increased resistance to blackleg, a dangerous disease caused mainly by *Leptosphaeria maculans*. In this study, we aimed to correlate the field resistance of selected *Brassica* hybrids to blackleg with chromosomal structure revealed by Fluorescence *in situ* hybridization. Tested genotypes varied in the number of chromosomes and rDNA signals. The greatest variation was observed for A1-type chromosomes. Field evaluation also revealed significant differences in *L. maculans* resistance. Performed analyses allowed to distinguish three *B. napus* × *Brassica fruticulosa* genotypes in which variable patterns of chromosomal structure might be connected to field resistance. However, a more thorough study, including the detection of all A-genome chromosomes, is required.

## 1 Introduction

The Brassicaceae family consists of many agronomically important species, that is, oil plants (rapeseed, oilseed, mustard) and vegetables (mustard cabbage, broccoli, cauliflower), as well as wild and noncultivated species. The natural genetic variability among the Brassicaceae species results in their richness in biotic and abiotic stress resistance genes [1]. Selected genotypes can act as a source of valuable traits which can be transferred through interspecific hybridization [2,3].

Rapeseed (*Brassica napus* L.), one of the most important sources of vegetable oil in the world [4], is an amphidiploid originating from diploid species *Brassica rapa* L. and *Brassica oleracea* L. Interspecific hybridization with species of Brassicaceae may result in broadening *B. napus* gene pool with useful characteristics such as resistance to *Leptosphaeria maculans*, the causal agent of blackleg. This disease, distributed worldwide, can lead to a substantial yield loss of  – up to 60% in favourable conditions [5]. Breeding of resistant cultivars is an environmentally friendly and reliable method of controlling blackleg disease [6]. Two types of resistance to *L. maculans* have been discovered: seedling resistance controlled by single major genes (R genes) and adult plant resistance conferred by multiple minor genes (QTL, quantitative trait loci) [7]. Considering the high evolutionary potential of *L. maculans*, low genetic diversity of rapeseed, as well as reported and predicted breakdowns of qualitative resistance in *B. napus*, it seems crucial to introduce new sources of resistance other than rapeseed [8–10]. Effective resistance genes can be found among several species of Brassicaceae, for example, *Arabidopsis thaliana, B. elongata, B. fruticulosa, B. juncea, B. carinata, B. nigra*, and *Raphanus sativus* [11–13]. That is why interspecific crosses of *B. napus* with its relatives may lead to transferring of the blackleg resistance genes to rapeseed, as previously reported [14–16]. For example, Rahman et al. [17] crossed susceptible cultivar Westar with resistant *B. carinata* accession, which resulted in obtaining BC_2_S_3_ DH population resistant to pathotype PG2 of *L. maculans*.

To confirm hybridity and verify the genetic variation at the chromosomal level, genotypes obtained by interspecific crossing may be characterized by methods of *in situ* hybridization. Genomic *in situ* hybridization (GISH) is used primarily to identify parental genomes in allopolyploid species. The results of the GISH analysis allow the demonstration of intergenomic structural rearrangements (translocations between individual ancestral genomes) [18–20] and provide information on the similarities between the DNA of related species [21]. However, Hasterok et al. [22] observed nonspecific probes hybridization while analysing the A and C genomes of *B. napus*, which might have been caused by the high homology of the aforementioned genomes. As the lack of entire chromosome arms painting limits the use of GISH technique in *Brassica*, another method of chromosome recognition should be applied. Fluorescence *in situ* hybridization (FISH) is a commonly used technique for analysing the structure of the plant genome. The use of rDNA sequences in the analysis of *Brassica* species enables the recognition of selected chromosomes (i.e., carrying rDNA sequences) and consequently gives deeper insight into genome structure and composition [23]. The use of the 5S rDNA and 35S rDNA sequences as probes in the FISH technique enables the identification of the A1, A3, A5/A6/A9, and A10 chromosomes in the *B. rapa* genome, C4, C7, and C8 in the *B. oleracea* genome, as well as B4, B5, and B6/7 in the genome of *B. nigra* [24].

This study aimed to correlate the field resistance of chosen *Brassica* hybrids to *L. maculans* with chromosomal structure revealed by FISH. The comparison of field evaluation with cytogenetics study allows assessing if the presence or absence of certain chromosome regions is directly connected to plants' response to blackleg disease. The use of FISH technique will additionally allow tracking chromosome rearrangements based on various rDNA loci patterns found in hybrid parental forms.

## 2 Materials and methods

The F_1_ generation of hybrids was developed at the Department of Genetics and Plant Breeding (Poznań University of Life Sciences) by performing crosses between *B. napus* (maternal species) and various Brassicaceae genotypes (paternal species) with known blackleg resistance level. The hybrids were developed using *in vitro* techniques. Next, chosen genotypes were self-pollinated to obtain F_2_ plants. The following interspecific hybrids of F_2_ generation were used as research material: *B. napus* × *B. rapa* ssp. *pekinensis, B. napus* × *B. rapa* ssp. *trilocularis, B. napus* × *B. rapa* ssp. *chinensis, B. napus* × *B. fruticulosa, B. napus* × *Brassica carinata, B. napus* × *Brassica juncea.* Field evaluation was performed to assess the level of hybrids resistance to *L. maculans* followed by FISH technique to analyse the chromosome architecture of chosen hybrids.

### 2.1 FISH

Seeds of studied genotypes were germinated in the dark for 3–4 days. Freshly cut seedlings’ roots were treated with 2 mM 8-hydroxyquinoline under the following conditions: 1 h at 4°C and 2 h at room temperature in the dark. Roots were fixed in ethanol–glacial acetic acid mixture (3:1) and stored at −20°C. Fixed plant material was macerated for 70–90 min depending on the genotype in an enzyme mixture consisting of 20% pectinase (Sigma), 1% cellulase (Calbiochem), and 1% cellulase ‘Onozuka R-10’ (Serva). Next, single root tips were squashed on the glass slide in 10 µL of 45% acetic acid. After a preliminary assessment of prepared slides, coverslips were removed by liquid nitrogen freezing and stored at 4°C until needed.

Two probes, 5S and 35S rDNA, were used in this study. The 5S rDNA sequence was derived from *Triticum aestivum* genome. The whole sequence (410 bp) was isolated from the plasmid vector with the use of QIAprep Spin Miniprep Kit (Qiagen), and labelled with rhodamine using PCR method under the following conditions: 94°C × 60 s, 35 cycles (94°C × 40 s, 55°C × 40 s, 72°C × 60 s), 72°C × 5 min. 35S rDNA is a 2.3kbp long fragment derived from *A. thaliana*. Plasmid vector sequence was isolated using QIAprep Spin Miniprep Kit (Qiagen) and labelled with digoxygenin by nick-translation (Nick Translation Kit, Sigma-Aldrich) under following conditions: 15°C × 95 min, 60°C × 10 min.

The double-target FISH procedure was carried out according to Hasterok et al. [22]. For every combination, five genotypes were assessed. The hybridization mixture consisted of 50% de-ionised formamide, 10% dextran sulphate, 2× SSC buffer, 10% SDS, SSS (salmon sperm blocking DNA), 5S and 35S rDNA probes. To detect digoxygenin labelled probe, FITC-conjugated anti-digoxigenin antibodies were used (Sigma-Aldrich). Chromosomes were later counterstained with DAPI (4,6-diamidino-2-phenylindole). Microscope slides were examined with epifluorescence microscope BX-61 (Olympus) equipped with XM10 monochrome camera (Olympus). Captured pictures were further analysed and processed with Micrografx Picture Publisher 10.0 software (Corel Corporation) and Adobe Photoshop software (Adobe). For every genotype we assessed the somatic number of chromosomes, the number of marker chromosomes, and the number of marker chromosome pairs. The chromosomes were identified according to nomenclature established by Xiong and Pires [24].

### 2.2 Resistance to *L. maculans*

Field evaluation was conducted in three subsequent years (2018, 2019, 2020) on the testing fields in Poznań University of Life Sciences experimental station Dłoń (51°41′23″N, 17°04′10″E) located 100 km south from Poznań, Poland. The whole experiment was set up in a completely randomized block design with five replications, and each single plot size was 10 m^2^ with a 0.30 m row distance and a sowing density of 60 seeds/m^2^. The field experiment in Dłoń was carried out on typical heavy soil of III quality class, with optimal agricultural practices for local agroecological conditions, and no artificial irrigation In crop seasons 2017/2018, 2018/2019, and 2019/2020, the weather conditions were typical for this region of Poland. The seasonal rainfall in Dłoń was 372 mm in 2018, 393 mm in 2019, and 405 mm in 2020, whereas the mean annual temperatures in 2018, 2019, and 2020 were 10.8, 11.1, and 10.5°C respectively. The pre-sowing fertilization on experimental plots consisted of ammonium phosphate (200 kg/ha) and ammonium nitrate (100 kg/ha). Four fertilizers were used during vegetation: Saletrosan 26 (300 kg/ha), ammonium nitrate (200 kg/ha), magnesium sulphate and ADOB Bor, containing borum and nitrogen. No fungicides and pesticides were used on the testing fields. Disease severity was assessed once a year in autumn (BBCH 11–14, leaf development) on five genotypes per combination, according to a percentage scale (Table 1). Whole plants were used to assess the plants' resistance. The average values from ten replications were calculated for each genotype in every combination.

**Table 1:** Disease severity percentage scale according to visual symptoms observed on whole plants

Percentage scale	Disease symptoms	Resistance level
0	No diseased tissue visible	Highly resistant
5	Lesions occupy 5% of the roots and leaves surface	Resistant/highly resistant
10	Lesions occupy 10% of the roots and leaves surface	Resistant
20	Lesions occupy 20% of the roots and leaves surface	Moderately resistant/resistant
25	Lesions occupy 25% of the roots and leaves surface	Moderately resistant
50	Lesions occupy 50% of the roots and leaves surface	Moderately susceptible/moderately resistant
75	Lesions occupy 75% of the roots and leaves surface	Moderately susceptible
90	Lesions occupy 90% of the roots and leaves surface	Susceptible/moderately susceptible
100	Dead plant	Susceptible

### 2.3 Statistical analysis

The normality of the distribution of the studied trait (*L. maculans* infestation) was tested using Shapiro–Wilk’s normality test [25]. Two-way analysis of variance (ANOVA) was carried out to determine the effects of combinations and years as well as combination × year interaction on the variability of infestation. The mean values and standard deviations of infestation were calculated. The Fisher’s least significant differences (LSDs) were calculated for infestation and on this basis, homogeneous groups were determined. The relationships between particular years were assessed based on Pearson’s correlation for infestation. These relationships were presented in heatmaps. One-way ANOVA was carried out to determine the effect of genotypes on the variability of infestation, independently for each combination. All analyses were conducted using the GenStat 18th edition statistical software package.

## 3 Results

### 3.1 FISH

**Figure 1:**
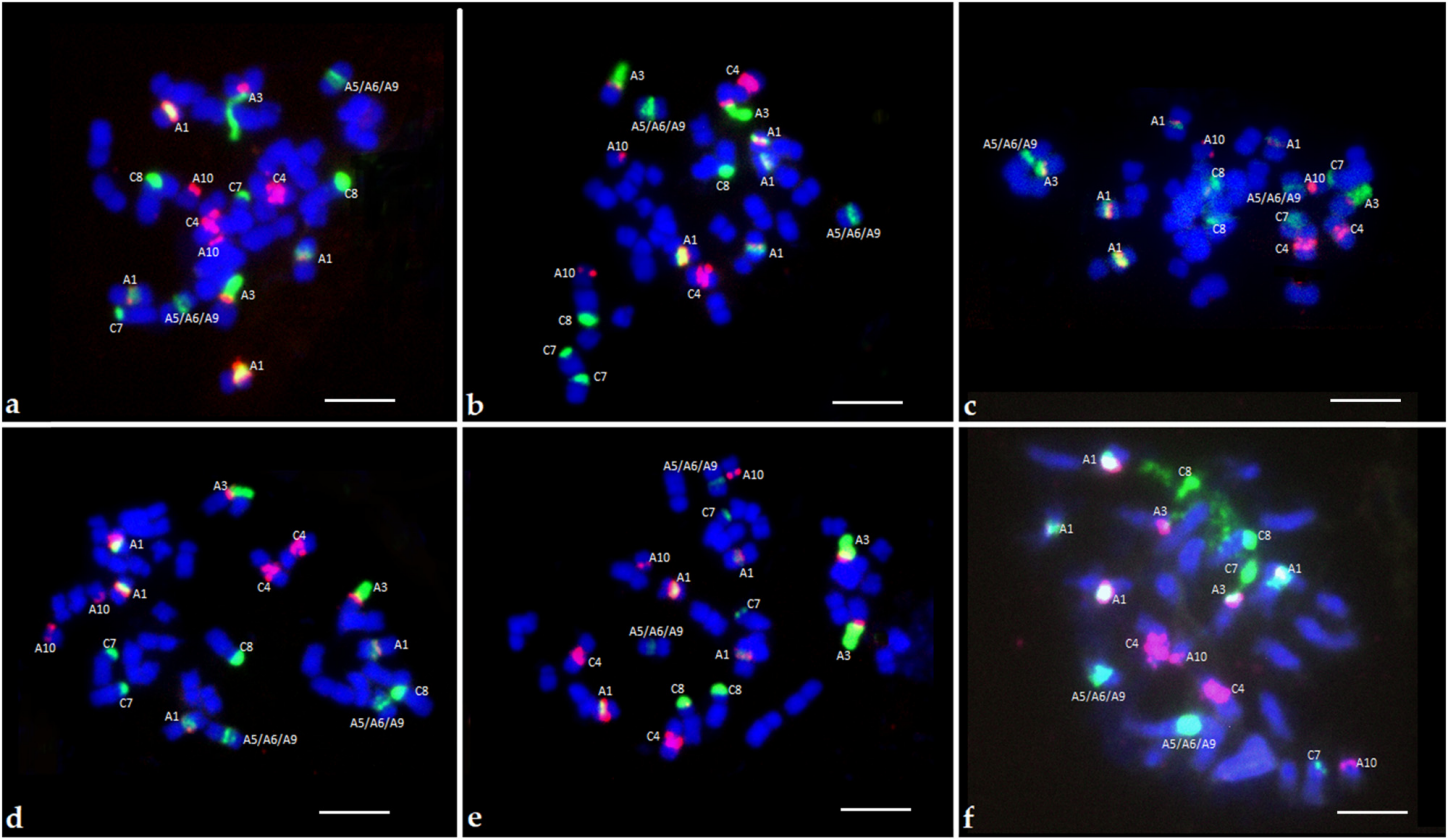
Identification of marker chromosomes in selected *Brassica* hybrids using 5S rDNA (red) and 35S rDNA (green) probes. (a) *B. napus* × *B. rapa* ssp. *pekinensis* genotype 2, (b) *B. napus* × *B. rapa* ssp. *trilocularis* genotype 2, (c) *B. napus* × *B. rapa* ssp. *chinensis* genotype 5, (d) *B. napus* × *B. fruticulosa* genotype 1, (e) *B. napus* × *B. carinata* genotype 4, (f) *B. napus* × *B. juncea* genotype 3. Scale bars represent 5 µm.

The FISH technique was used to assess the chromosomal composition of newly generated hybrids that were created by crossing between allotetraploid *B. napus* with other allopolyploid *Brassica* species, such as *B. carinata* and *B. juncea*, as well as with diploid *B. fruticulosa* and *B. rapa* (ssp. *pekinensis*, *trilocularis*, and *chinensis*). Detailed results are presented in Table 2. Generally, performed analyses allowed to assess the general number of chromosomes and distinguish the chromosomal types that are characteristic for A and C genome (Figure 1).

**Table 2:** Number of detected chromosomes, rDNA signals, and marker chromosomes revealed by FISH analysis

						Number of marker chromosomes									
Combination	Genomic constitution	No. of genotype	2*n*	5S rDNA loci number	35S rDNA loci number	A1	A3	A10	A5/A6/A9	C4	C7	C8	B4	B5	B6/7
*B. napus* × *B. rapa* ssp. *pekinensis*	AACC × AA	1	38	10	12	4	2	2	2	2	2	2	—	—	—
		2	38	10	12	4	2	2	2	2	2	2	—	—	—
		3	38	12	14	6	2	2	2	2	2	2	—	—	—
		4	38	10	12	4	2	2	2	2	2	2	—	—	—
		5	38	12	14	6	2	2	2	2	2	2	—	—	—
*B. napus* × *B. rapa* ssp. *trilocularis*	AACC × AA	1	38	11	13	5	2	2	2	2	2	2	—	—	—
		2	38	10	12	4	2	2	2	2	2	2	—	—	—
		3	38	11	13	5	2	2	2	2	2	2	—	—	—
		4	38	11	13	5	2	2	2	2	2	2	—	—	—
		5	38	10	12	4	2	2	2	2	2	2	—	—	—
*B. napus* × *B. rapa* ssp. *chinensis*	AACC × AA	1	38	10	12	4	2	2	2	2	2	2	—	—	—
		2	38	10	12	4	2	2	2	2	2	2	—	—	—
		3	38	10	12	4	2	2	2	2	2	2	—	—	—
		4	38	10	12	4	2	2	2	2	2	2	—	—	—
		5	38	10	12	4	2	2	2	2	2	2	—	—	—
*B. napus* × *B. fruticulosa*	AACC × FF	1	36	10	12	4	2	2	2	2	2	2	—	—	—
		2	36	12	13	6	2	2	2	2	1	2	—	—	—
		3	36	10	12	4	2	2	2	2	2	2	—	—	—
		4	36	10	12	4	2	2	2	2	2	2	—	—	—
		5	34	8	10	2	2	2	2	2	2	2	—	—	—
*B. napus* × *Brassica carinata*	AACC × BBCC	1	38	10	12	4	2	2	2	2	2	2	0	0	0
		2	38	10	12	4	2	2	2	2	2	2	0	0	0
		3	38	10	12	4	2	2	2	2	2	2	0	0	0
		4	38	10	12	4	2	2	2	2	2	2	0	0	0
		5	38	10	12	4	2	2	2	2	2	2	0	0	0
*B. napus* × *Brassica juncea*	AACC × AABB	1	37	10	12	4	2	2	2	2	2	2	0	0	0
		2	37	10	12	4	2	2	2	2	2	2	0	0	0
		3	37	10	12	4	2	2	2	2	2	2	0	0	0
		4	37	10	12	4	2	2	2	2	2	2	0	0	0
		5	37	10	12	4	2	2	2	2	2	2	0	0	0

The first group of hybrids – *B. napus* × *B. rapa* ssp. *pekinensis* was invariable in the number of observed chromosomes: all five genotypes had 38 chromosomes. However, the studied hybrids differed in the number of detected rDNA signals. Two types of rDNA patterns were observed: 10 5S rDNA loci and 12 35S rDNA loci as well as 12 5S rDNA loci and 14 35S rDNA loci. In the first group, the following were observed: two A3 chromosomes, four A1 chromosomes, two A10 chromosomes, two chromosomes of the group A5/6/9, two C4 chromosomes, two C7 chromosomes and two C8 chromosomes. In the second group, a similar pattern of individual marker chromosomes was observed, except for A1 chromosomes the number of which was six.

All studied *B. napus* × *B. rapa* ssp. *trilocularis* genotypes had 38 chromosomes. Among tested hybrids, we observed two genotypes containing ten 5S rDNA signals and twelve 35S rDNA signals, which allowed to identify two A3 chromosomes, four A1 chromosomes, two A10 chromosomes, two A5/6/9 chromosomes and two C4 chromosomes, two C7 chromosomes and two C8 chromosomes. For three remaining genotypes, we revealed eleven 5S rDNA signals and thirteen 35S rDNA signals, which enabled us to recognize eleven chromosomes from the A genome – one additional A1 chromosome, and six chromosomes of the C type.

*B*. *napus* × *B*. *rapa* spp. *chinensis* hybrids did not vary in the total number of chromosomes. All five genotypes with 38 chromosomes had ten 5S rDNA loci, twelve 35S rDNA loci, and the same number of chromosome types: four A1 chromosomes and two chromosomes of each of A3, A10, A5/A6/A9, C4, C7, C8 chromosome types.

In the *B. napus* × *B. fruticulosa* group, a variable number of chromosomes was observed. Genotypes with 36 chromosomes predominated, but one genotype with 34 chromosomes was also observed. Two patterns of rDNA loci were detected in the group with 36 chromosomes: ten 5S rDNA loci and twelve 35S rDNA loci, as well as twelve 5S rDNA loci and thirteen 35S rDNA loci. In a genotype with 34 chromosomes, eight 5S rDNA loci and ten 35S rDNA loci were observed. A constant number of chromosomes was identified in all genotypes of a given combination: two A3 chromosomes, two A10 chromosomes, two A5/6/9 chromosomes, two C4 chromosomes, and two C8 chromosomes. A variable number of chromosomes was revealed for A1 type (two, four, and six chromosomes), and C7 type (one and two chromosomes).

For *B. napus* × *B. carinata* hybrids, the total number of chromosomes was consistent – 38 chromosomes were identified in all genotypes. The rDNA loci pattern was constant for all studied genotypes (ten 5S rDNA loci and twelve 35S rDNA loci). Moreover, the number of chromosomes observed was also the same for all individuals: four A1 chromosomes, two A3 chromosomes, two A10 chromosomes, two A5/A6/A9 chromosomes, two C4 chromosomes, two C7 chromosomes, and two C8 chromosomes. Genome B derived chromosomes were not detected.

A constant number of chromosomes of 37 chromosomes and a constant number of 5S and 35S rDNA loci, that is, 10 and 12 rDNA loci was identified in *B. napus* × *B. juncea* hybrids. The same number of individual types of marker chromosomes (carrying rDNA sequences) was observed in five genotypes: four A1 chromosomes, two A10 chromosomes, two A3 chromosomes, two chromosomes from the A5/6/9 group, two C4 chromosomes, two C7 chromosomes, and two C8 chromosomes. No chromosomes derived from the B genome were detected.

### 3.2 Resistance to *L. maculans*

Field evaluation was used to determine the level of resistance to blackleg of studied genotypes in three following years. The ANOVA analysis revealed that the effect of the combination, year, combination × year was statistically significant for infestation level (Table 3). Table 4 presents the mean values of infestation in three years for six analysed hybrid combinations. In general, the infestation level varied between studied combinations in 2018, 2019, and 2020, which allowed distinguishing groups of the most resistant (least infested) plants in each year. The highest level of infestation (16.866) was observed for *B. napus* × *B. juncea* combination in 2019 when the lowest level of *L. maculans* damage (0.00) was observed in 2019 and 2020 for *B. napus* × *B. fruticulosa*. Moreover, the latter combination belonged to the statistically best group in all three consecutive years.

**Table 3:** Mean squares (ms) from analysis of variance for *L. maculans* infestation

	d.f.^1^	ms
Combination	5	224.74***
Year	2	221.72***
Combination × year	10	57.27***
Residual	72	11.48

^1^Degrees of freedom ***P < 0.001.

**Table 4:** Mean values and standard deviations (sd) for *L. maculans* infestation for combinations and years of study

Year	2018		2019		2020		2018–2020	
Combination	Mean	sd	Mean	sd	Mean	sd	Mean	sd
*B. napus* × *B. rapa* ssp. *pekinensis*	15.998a	2.042	7.732bc	4.037	7.866a	2.233	10.532a	4.825
*B. napus* × *B. rapa* ssp. *trilocularis*	7.93bc	0.548	6.066cd	1.361	5.132ab	2.919	6.376b	2.121
*B. napus* × *B. rapa* ssp. *chinensis*	12.866ab	5.007	10.4b	3.782	5.866a	5.709	9.711a	5.438
*B. napus* × *B. fruticulosa*	4.666c	0.942	0e	0	0c	0	1.555c	2.332
*B. napus* × *B. carinata*	3c	3	3.666d	1.248	0.532c	0.728	2.399c	2.262
*B. napus* × *B. juncea*	8.53bc	8.556	16.866a	3.639	2.132bc	0.181	9.176a	7.981
Average	8.832A	6.017	7.455A	5.974	3.588B	3.885		
LSD0.05	Combination: 2.466; year: 1.744; combination × year: 4.271							

Values with different letters in columns are significantly different.

The infestation level was also compared between genotypes of certain combinations (Table 5). For this purpose, we calculated the mean values of infestation from three years for every genotype. For most studied individuals, no significant differences in infestation were observed, however for *B. napus* × *B. fruticulosa* we were able to distinguish three genotypes with higher resistance to blackleg (infestation level 1.33). From all analysed plants, *B. napus* × *B. rapa* ssp. *pekinensis* genotype number 5 showed the highest level of infestation (13.67), and the highest resistance (1.00) was noted for *B. napus* × *B. carinata* genotype number 2.

**Table 5:** Mean values and standard deviations (sd) for *L. maculans* infestation for genotypes from particular combinations

Genotype	*B. napus* × *B. rapa* ssp. *Pekinensis*		*B. napus* × *B. rapa* ssp. *trilocularis*		*B. napus* × *B. rapa* ssp. *Chinensis*		*B. napus* × *B. fruticulosa*		*B. napus* × *B. carinata*		*B. napus* × *B. juncea*	
	Mean	sd	Mean	sd	Mean	sd	Mean	sd	Mean	sd	Mean	sd
1	7.33a	5.292	6.887a	1.709	9.777a	6.621	1.33b	2.309	2.11a	2.009	11.22a	7.728
2	8.55a	5.983	5.443a	2.693	10.443a	9.894	1.78ab	3.077	1a	1.732	12.44a	9.196
3	10.56a	4.717	6.553a	3.077	9.333a	2.309	2.00a	3.464	1.44a	1.503	9.22a	12.221
4	12.55a	3.67	7.553a	1.345	13.333a	2.082	1.33b	2.309	4.44a	2.876	6.55a	7.315
5	13.67a	4.619	5.443a	2.218	5.667a	3.215	1.33b	2.309	3a	2.646	6.44a	7.412
LSD0.05	8.94		4.178		10.35		0.66		4.033		16.31	
*F*-ANOVA	0.87		0.49		0.7		0.04		1.14		0.27	

Values with different letters in columns are significantly different.

## 4 Discussion

Performed FISH analyses allowed to successfully distinguish parental chromosomes in hybrids genomes in most studied combinations. However, the recognition of B genome chromosomes was not accomplished. Hasterok et al. [26] used 5S and 25S rDNA probes to identify genomes of various *Brassica* species. This approach allowed to distinguish eight out of sixteen B genome chromosomes in *B. nigra*, as well as 20 out 36 chromosomes in *B. juncea*, including eight B genome chromosomes. In our study, B type chromosomes would be expected to appear in *B. napus* × *B. carinata* and *B. napus* × *B. juncea* combination, although such chromosomes have not been detected. This result can be explained simply by the absence of B genome derived chromosomes in analysed hybrids. However, it may be also elucidated by a great similarity in chromosome morphology between A, B, and C genomes, including rDNA-bearing chromosomes, which may consequently lead to misinterpretation of rDNA signals and incorrect recognition.

In this study, we observed a variation in the number of chromosomes for one combination – *B. napus* × *B. fruticulosa*, as well as the variation in the number of detected 5S and 35S rDNA signals between genotypes for three combinations. This might be explained by irregular chromosome segregation, conversion of genes, or unequal crossing-over events [27]. The greatest variation was observed for A1 chromosomes – for *B. napus* × *B. rapa* ssp. *trilocularis* their number varied from 4 to 5, for *B. napus* × *B. rapa* ssp. *pekinensis* – from 4 to 6, and for *B. napus* × *B. fruticulosa* – from 2 to 6. A study of Sosnowska et al. [23] on resynthesized *B. napus* also indicates the A1 chromosomes as the most variable in their number.

Thirty-seven chromosomes were observed in the genotypes resulting from the crossing of *B. napus* and *B. juncea*. An odd number of chromosomes can reduce plant fertility due to cytogenetic instability during meiosis (incorrect chromosome pairing). Nevertheless, it is in line with expectations, considering the number of chromosomes found in the parental components – *B. napus* (2*n* = 38) and *B. juncea* (2*n* = 36). In the *B. napus* (2*n* = 38) × *B. carinata* (2*n* = 34) combination, 38 chromosomes were observed for all genotypes. A higher number of chromosomes than expected may be due to disturbances during the meiotic division.

It is known that the genomes of allopolyploids undergo dynamic changes, which result in intergenomic rearrangements [28]. The polymorphism of rDNA sequences observed in this study might be caused by structural rearrangements. The occurrence of such events would not be surprising since considerable homology exists between A and C genome. Heneen et al. [29] observed pairing of homologous chromosomes of *B. rapa* and *B. oleracea* genomes in the monosomic alien addition lines, which confirms the close relationship of species. Translocations may also occur in *B. napus* genome [30,31].

Our research proved that *Brassica* species can be a good source of resistance to blackleg disease – especially the *B. napus* × *B. fruticulosa* genotypes, which showed a low and stable level of infestation in all three years of the study. Moreover, resistance genes may be successfully transferred to hybrid species, and possibly extend *B. napus* gene pool. As stated before, interspecific crossing can give rise to new enhanced rapeseed cultivars with improved characteristics [32]. Nevertheless, the use of relative species of rapeseed as a source of *L. maculans* resistance might be dangerous due to the possibility of rapid overcome of major gene resistance in various cruciferous hosts. This event has been described in a study by Li et al. [33], in which several Brassicaceae species have been analysed in terms of their response to inoculation by *L. maculans* isolates in Australia. The pathogen was able to overcome the resistance of most tested genotypes, which means that close attention and carefulness should be taken when introducing new sources of resistance to *L. maculans* into breeding programs.

*L. maculans* resistance has been identified in several *Brassica* species including A-genome, B-genome, C-genome, and AC-genome species [14]. It would be expected that lack or addition of certain chromosomes in studied hybrids influence the level of resistance to blackleg, however for most studied individuals such effect was not observed, as for five out of six combinations, no significant differences in resistance level between genotypes were noted. Detailed study of *B. napus* × *B. fruticulosa* combination, the only one with the statistically important variation of *L. maculans* resistance might give more information regarding the correlation of this trait with chromosomal structure. The most resistant genotypes of the aforementioned hybrid combination differed in the number of detected chromosomes and rDNA loci, two of them containing 36 chromosomes, and one containing 34 chromosomes. The latter was also characterized by a lower number of rDNA signals. The variable pattern of chromosomal structure detected in *B. napus* × *B. fruticulosa* genotypes might be connected to blackleg resistance, nevertheless, it should be taken into consideration that not all resistance-bearing chromosomes were identified in this study. Major *L. maculans* resistance genes have been mapped on four A-genome chromosomes: A1, A10, A7, and A2 [8]. The latter two, which contained six resistance genes were not a part of our FISH experiment. Additional detection of previously unrecognized chromosomes combined with continued resistance assessment could provide more information about the connection between chromosomal structure and blackleg resistance, and give deeper insight into *Brassica* hybrids' genomic composition.

## 5 Conclusion

1. The use of 5S rDNA and 35S rDNA probes allow to successfully identify parental genomes in chosen *Brassica* hybrid combinations.
2. The observed variable number of chromosomes and the number of 5S and 35S rDNA loci in the genomes of the *Brassica* hybrid plants may be due to transposition or deletion within the chromatin containing rDNA sequences, elimination of marker chromosomes during meiosis, or deletion or amplification of rDNA loci.
3. Selected *Brassica* hybrids can be a good source of *L. maculans* resistance in rapeseed breeding programs.
4. Three *B. napus* × *B. fruticulosa* genotypes with highest resistance to *L. maculans* varied in chromosome number and the number of detected rDNA loci. However, a more thorough study, including the detection of all A genome chromosomes, is required.
